# The role of folded fibular flap in patients’ reconstruction of mandibular defects: a retrospective clinical study

**DOI:** 10.1038/s41598-021-03331-7

**Published:** 2021-12-13

**Authors:** Ning Gao, Kun Fu, Jinghua Cai, Hao Chen, Wei He

**Affiliations:** Department of Oral and Maxillofacial Surgery, First Affiliated Hospital of Zhengzhou University, Zhengzhou, 450052 China

**Keywords:** Psychology, Diseases, Oncology

## Abstract

This study has analyzed 41 patients with mandibular ameloblastoma who underwent a partial mandibulectomy and reconstruction by folding the free fibular flap. In the preoperative and postoperative (6 months and 24 months after surgery), the Quality of Life (QOL) of these patients was assessed by using the University of Washington Quality of Life Questionnaire (UW-QOL) and the medical outcome study short form-36 (SF-36) questionnaires. SPSS 20.0 statistical software was used to conduct statistical analysis on the base data of the two groups of patients. Independent sample *t* test was conducted for sf-36 and UW-QOL scores at two time points in each group. The SF-36 survey showed that body pain (54.54 ± 8.10), general health (55.27 ± 7.54), and health changes (58.29 ± 9.60) decreased significantly at 6 months after surgery, but the mean score at 24 months after surgery all exceeded the preoperational level. At 24 months after the surgery, the vitality (80.41 ± 3.74), social function (81.61 ± 4.07), emotional role (82.39 ± 4.07), psychological health (81.66 ± 4.37) and total score (704.00 ± 31.53) all returned to the preoperative level, which was statistically significant compared with 6 months after surgery. However, there was no significant difference compared with the preoperative level. The UW-QOL survey showed that chewing (56.68 ± 7.23), speech (54.54 ± 7.7) and taste (62.29 ± 10.15) have significantly changed at 6 months after the surgery, and the difference was statistically significant at 24 months after surgery. Saliva generation decreased slightly (80.76 ± 3.35) at 6 months after surgery, but quickly returned to the preoperative level (81.59 ± 4.06). The total score of the patients almost recovered to the preoperative level at 24 months after surgery. The folded the fibular flap can not only repair the defects of soft tissue and bone tissue, but also restore the height of the alveolar ridge to, avoid the imbalance of crown and root ratio after implantation and reduce the occurrence of peri-implant inflammation, so that a true functional reconstruction can be realized.

## Introduction

Since 1989 when Hidalgo first reported the success of using the free fibular flap to repair mandibular defects, the clinical application of this surgical technique to repair damage associated with tumor removal has seen further improvement^1^.With the constant improvement of the microsurgical technique^2^, as well as 3D printing^3^, the upgrade of free fibular flap surgical technique offers increasingly extensive applications in the repair of mandibular defects^4^. Ameloblastoma is a common cause of mandibular defects. The free fibula combined with dental implant technology for functional reconstruction of the mandible can effectively restore the patient’s facial shape and occlusal relationship. However, the biggest disadvantage, the height of the reconstructed mandible cannot meet the needs of implant repair^5^. The mucosal scar on the surface of the single fibular is thick^6^, which not only increases the difficulty of imprinting^7^, but also easily produces peri-implant inflammation^8^. The folded fibular flap perfectly restores the height of the alveolar ridge, avoiding the imbalance of the coronal root ratio in implant repair.

With the transformation of the biomedical model into the bio-psycho-social medical model, the criteria for evaluating the effectiveness of tumor treatments, are no longer restricted purely to survival rate and biological indicators such as organ function reconstruction^9^, but now also encompass improvements made to the patients’ QOL^10^. In this study, The UW-QOL and the SF-36 questionnaires were used to evaluate and analyze the QOL of patients with tumor removal related tissue defects, repaired with the folded fibular flap technique. This study provides a basis for the formulation of a comprehensive treatment plan, the selection of surgical methods and repair methods, the postoperative functional rehabilitation and the possible psychological interventions resulting from the use of the fibular flap in the reconstruction of maxillofacial defects.

## Results

All 41 cases undergoing this procedure survived. 39 patients healed in the first stage of healing, and two developed infection, which healed after antibiotic treatment and local dressing changes. The shaping time for the fibular was 43–90 min, with an average of (61 ± 12) min, and the total operation time was 314–406 min, with an average of (356 ± 22) min. There were 40 cases of primary healing in the donor area and 1 case of scar healing after partial skin necrosis. Patients were able to return to their normal preoperative functional status at 6 weeks. The patients self-reported that there were no obvious abnormalities in the lower limb function compared with the preoperative level. Patients were questioned as to differences in sensation motor function etc. between the 2 limbs and preop status and this was also negative. There was no tenderness in the temporomandibular joint area and the degree of opening was 3.1–3.9 cm in 39 patients. Implants were implanted at 6–9 months after surgery, and the porcelain crown was repaired 3–4 months later. The implant bone was well combined, the occlusal relationship was restored, and the functions of speech, chewing and swallowing were returned to normal. All patients recovered the natural curvature, height and occlusal relationship of the defect area of the mandible, and no obvious abnormalities were found in the operative area. There were no obvious complications in the donor area, the lower limbs could bear weight, walking was not affected, and the donor area of the legs had no obvious dysfunction. There was no obvious peri-implantitis at the follow-up visit. All 41 patients completed the survey, and the completion rate was 100%. The general situation is shown in Table 1.

**Table 1:** Details of patients.

Variables	Cases n (%)
**Age (years)**	
< 50	29 (70.73)
50 and over	12 (29.27)
**Sex**	
Male	26 (63.41)
Female	15 (36.59)
**Primary sites of tumor**	
Body	19 (46.34)
Body, angle	12 (29.27)
Body, angle, ramus	10 (24.39)
**Clinical type**	
Solid	32 (78.05)
Unicystic	9 (21.95)
**Radiographic appearance**	
Unilocular	14 (34.15)
Multilocular	27 (65.85)
**Repair range**	
Unilateral body	10 (24.39)
Unilateral body and ramus	28 (68.29)
Bilateral body	3 (7.32)
**Recipient artery**	
Facial artery	37 (90.24)
Superior thyroid artery	4 (9.76)
**Recipient vein**	
Unidentified branch of internal jugular vein	32 (78.05)
External jugular vein	9 (21.95)

### SF-36 survey results

The specific score of SF-36 is shown in Table 2. The Physical role decreased significantly after the surgery. Although there was a significant improvement in the 24 months after the surgery compared with the first 6 months after the surgery (t = − 5.134, p = 0.000), it still could not return to the preoperative level (t = 11.685, p = 0.003). Body pain (54.54 ± 8.10), general health (55.27 ± 7.54), and health changes (58.29 ± 9.60) decreased significantly at 6 months after surgery, but the mean score at 24 months after surgery all exceeded that before surgery.

**Table 2:** Means of scores of items and scales of SF-36 Questionnaire.

Domains	Mean ± standard deviation			Comparison of t value (P value) at different time		
	Preoperative	6 months	24 months	Preoperative and 6 months	Preoperative and 24 months	6 months and 24 months
Physiological function	67.88 ± 5.77	48.34 ± 12.05	69.54 ± 7.29	9.365 (0.000)	− 9.635 (0.002)	− 1.142 (0.282)
Physical role	80.05 ± 9.41	44.90 ± 16.98	59.41 ± 6.27	11.594 (0.001)	11.685 (0.003)	− 5.134 (0.000)
Body pain	72.00 ± 10.70	54.54 ± 8.10	82.44 ± 12.90	8.333 (0.001)	− 3.989 (0.000)	− 11.727 (0.000)
General health	72.85.4 ± 9.50	55.27 ± 7.54	83.17 ± 12.07	9.285 (0.018)	− 4.300 (0.053)	− 12.554 (0.000)
Vitality	82.44 ± 5.20	77.78 ± 6.60	80.41 ± 3.74	3.552 (0.366)	2.024 (0.163)	− 2.224 (0.036)
Social function	84.00 ± 5.19	76.07 ± 7.40	81.61 ± 4.07	5.616 (0.066)	2.321 (0.107)	− 4.197 (0.002)
Emotional role	82.78 ± 4.23	74.83 ± 6.66	82.39 ± 4.07	6.457 (0.006)	0.426 (0.998)	− 6.204 (0.005)
Psychological health	80.05 ± 4.01	72.15 ± 7.97	81.66 ± 4.37	5.674 (0.000)	− 1.738 (0.415)	− 6.701 (0.000)
Health change	77.95 ± 3.81	58.29 ± 9.60	83.37 ± 4.83	12.188 (0.000)	− 5.637 (0.037)	− 14.939 (0.002)
Total	700.00 ± 32.10	557.41 ± 49.24	704.00 ± 31.53	15.533 (0.000)	− 0.569 (0.893)	− 16.053 (0.000)

At 24 months after the operation, vitality (80.41 ± 3.74), social function (81.61 ± 4.07), emotional role (82.39 ± 4.07), psychological health (81.66 ± 4.37) and total score (704.00 ± 31.53) all returned to the preoperative level, which was statistically significant compared with the 6 months after the operation, but there was no significant difference compared with the preoperative level.

### UW-QOL survey results

The specific score of UW-QOL is shown in Table 3. The items with significant changes after 6 months were chewing (56.68 ± 7.23), speech (54.54 ± 7.7) and taste (62.29 ± 10.15), which have significantly improved at 24 months after the surgery and the difference was statistically significant. Saliva generation decreased slightly (80.76 ± 3.35) at 6 months after surgery, but quickly returned to the preoperative level (81.59 ± 4.06).

**Table 3:** Means of scores of items and scales of UW-QOL questionnaire.

Domains	Mean ± standard deviation			Comparison of t value (P value) at different time		
	Preoperative	6 months	24 months	Preoperative and 6 months	Preoperative and 24 months	6 months and 24 months
Swallowing	64.17 ± 3.47	61.51 ± 3.61	64.78 ± 3.39	3.401 (0.982)	− 0.804 (0.791)	− 4.225 (0.787)
Speech	63.76 ± 2.91	54.54 ± 7.7	63.9 ± 2.9	9.802 (0.000)	− 0.223 (0.596)	− 11.675 (0.000)
Taste	81.92 ± 4.33	62.29 ± 10.15	74.66 ± 9.83	11.385 (0.000)	4.331 (0.001)	− 5.601 (0.693)
Saliva	81.46 ± 6.51	80.76 ± 3.35	81.59 ± 4.06	0.619 (0.004)	− 0.102 (0.056)	− 1.009 (0.114)
Total	359.68 ± 10.39	315.78 ± 20.25	354.12 ± 11.75	12.354 (0.000)	0.402 (2.271)	− 10.489 (0.001)

There was no significant change in the swallowing function after the surgery, and the total score of the patients almost recovered to the preoperative level at 24 months after the surgery (t = 2.271, p = 0.402).

## Discussion

The mandibular defects directly affect the facial features, masticatory and articulation functions of the patient^11^. This damage also restricts the daily life and social activities of the patients to different degrees, and indirectly affects the psychological state and social activities of the patients^12^. Traditional indicators for the evaluation of oral diseases do not consider the impact of diseases on the quality of life of patients. With the change of medical model, it has become a goal that surgeons pursue to ensure patients receive better QOL while prolonging the survival of tumor patients through medical intervention^13^.

**Figure 1:**
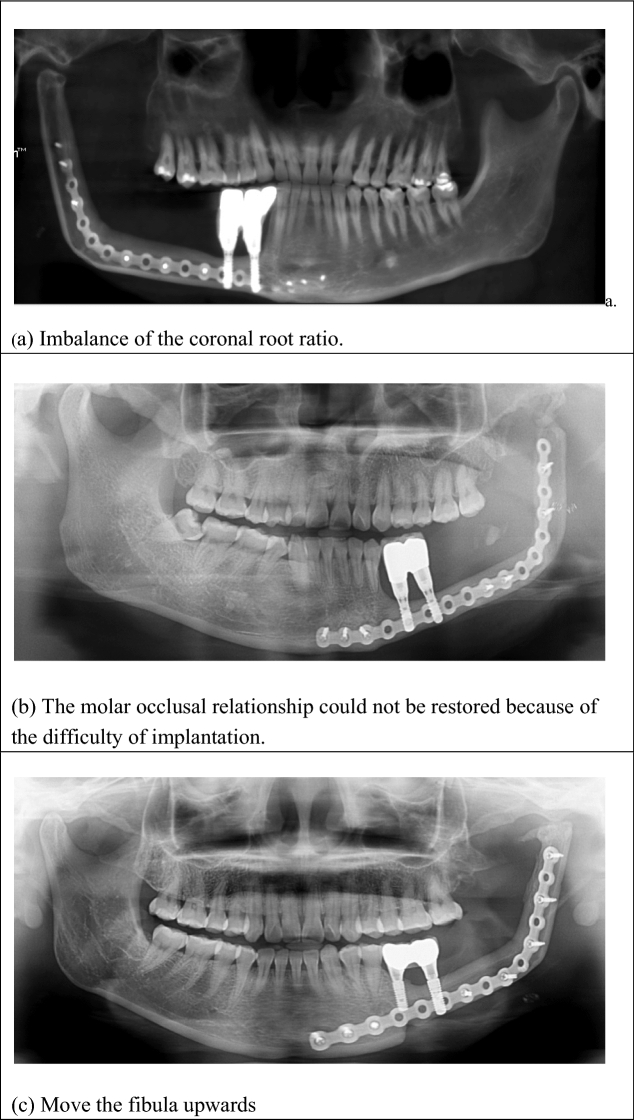
(**a**) The height of the reconstructed mandible cannot meet the needs of implant repair. (**b**) The mucosal scar on the surface of the single fibular is thick, which not only increases the difficulty of implanting and imprinting, but also easily produces peri-implant inflammation. (**c**) We can move the fibula upwards, but this may result in maxillofacial asymmetry.

With the constant improvement of the microsurgical technique, as well as 3D printing, the upgrade of free fibular flap surgical technique offers increasingly extensive applications in the repair of the mandibular defects^14^. It can repair not only bone tissue defects, but also soft tissue defects. When looking to repair the upper and lower jaw and adjacent tissue defects it is the tissue flap of choice. However, the biggest disadvantage when using the fibular flap is that the height is only 1.3–1.5 cm. Therefore, the height of the reconstructed mandible cannot meet the needs of implant repair. In addition, the mucosal scar on the surface of the single fibular is thick, which not only increases the difficulty of imprinting^15^, but also easily produces peri-implant inflammation (Fig. 1). Alternatively, the folded fibular flap perfectly restores the height of the alveolar ridge, avoiding the imbalance of the coronal root ratio in implant repair^16^. The thin crest mucosa reduces the incidence of peri-implant inflammation, which greatly improved the sample patient groups chewing function, language expression, and further increased the patient's confidence. All flaps of the selected cases in this group survived. This included the 2 patients presenting with flap crisis at 12–24 h after the operation which resulted in blocked venous reflux from poor drainage. The flap remained completely viable following efforts to improve drainage, which involved the removal of blood clots at the bottom of the mouth and microthrombus at the anastomosis^17^.

After fibular transplantation, more than 90% of the follow-up patients reported that the discomfort of lower limb pain, weakness, numbness and other discomfort gradually disappeared after 6 months, and the feeling and function of lower limb movement were basically normal, without affecting daily activities, which indicated that the flap resection of the donor area had almost no effect on the mobility of the patients.

The SF-36 results show that: (1) all functions of the patients recovered after the surgery, but the body still could not return to the preoperative level at 24 months after the surgery, the patients still felt slight discomfort in the lower limbs, itching in the scar, and even some patients still could not fully accept the fact that the fibular flap was placed in the maxillofacial region. (2) body pain, general health and health changes decreased significantly after the surgery, which was related to the recovery of the lower limbs of the patients. With the further exercise of function and the restoration of implant teeth, the average score at 24 months after the surgery was higher than the preoperational level, and the difference was statistically significant. (3) physiological function, vitality, social function, emotional role, mental health and the total score decreased significantly at 6 months after the operation, which was significantly related to the surgical trauma and the self-discomfort of the patients, but recovered to the preoperative level at 24 months after the operation.

UW-QOL results show that: (1) with the passage of time, the oropharyngeal function of the patients after the operation was significantly improved, especially after the implant was used to repair the dentition defect, the chewing and language of the patients were substantially changed. (2) some patients suffered from damage to the lingual nerve during the operation, postoperative tongue numbness and a decline in taste. This generally recovered 3 months following the operation. (3) since the submandibular gland of the affected side will be routinely removed during the operation, there will be a temporary decrease in the amount of saliva produced after the operation, but with the compensation of other glands, the patient’s symptoms decreased significantly. Results from the survey indicate that the impact on swallowing as a result of this surgery is not significantly adverse.

Emotional factors are the most influential issues for cancer patients^12^. We found that while the patient recovered in terms of physiological function, social function and family status, the emotional status could not return to the normal level at 6 months after the surgery^18^. The low scores of patients in mental health are largely related to the psychological reality of patients’ inability to accept jaw resection and their inability to adapt to changes in basic life functions such as speech and eating after surgery^19^. Patients are always worried about the recurrence of the tumor and the uncertainty of the future^20^, so they often experience symptoms such as irritability, fatigue, insomnia, pain, diarrhea and constipation^21–24^. This indicates that the emotional damage caused by the tumor to patients is more serious and long-lasting^25^. Patients think they are sicker than others and that their health is deteriorating^26^. Therefore, psychological and emotional rehabilitation treatment for patients is very important^27^. The psychological problems of cancer patients have aroused the attention of clinicians. While treating the patients’ diseases, the patients should be taught about the disease knowledge before the operation to alleviate their fear and anxiety about the unknown. After the operation, the discomfort of the oral, maxillofacial and lower limbs should be diagnosed and treated in a timely manner, and the patients should be encouraged to actively participate in social activities. At the same time, the survey shows that the encouragement and support of family members play a vital role in the physical and mental health of patients, and the love from family members makes it easier for patients to recover from the trauma of the disease. Therefore, the patient’s psychological discomfort and social disorder require not only the joint efforts of doctors and patients, but also the care and support of family members.

The surface of a single layer fibula is covered with thick scars and mucous membranes, which is prone to peri-implant inflammation. It is advised that peri-implantitis and mucositis after fibula repair were identified in 14.8% and 20.3% of surviving implants, respectively, at the 5- and 10-year follow-up^28^. The study also suggests that skin or connective tissue grafts seem to offer an aid to manage this problem. We have found in the clinic that the incidence of peri-implant inflammation after folding the fibula is relatively low. This is because the mucosa on the surface is thin, which facilitates the smooth neck of the implant to penetrate the gums and facilitates the self-cleaning of the implant neck. The 12-month clinical trials have shown that there is no significant difference between the restoration supported by a single short implant and the long implant^29,30^. Although the three-year clinical trial of 2018 supported the view mentioned above^31^, the seven-year clinical study of 2021concluded that the survival rate of short implants was significantly lower than that of conventional implants (87% vs. 100%)^32^. At present, there is no relevant research on the restoration of multiple missing teeth on the fibula. Therefore, we also need to make more efforts in the clinic to study whether the use of crown-to-root ratio in a single-layer fibula is not different from that of a folded fibula.

It is suggested that the folded fibula is the best repair and reconstruction plan for the defect of the middle part of the mandible, and it provides a good foundation for implant restoration^33^. Compared with the conventional one-strut fibula transplant, the “double-barrel” graft achieved more bone height and appreciably reduced the vertical distance to the occlusal plane. This technique creates better conditions for prosthetic rehabilitation. The folded fibula and iliac bone graft can better reconstruct the vertical height of fibula than vertical traction^34,35^.

The implant restoration after fibular transplantation is still in the exploratory stage. Therefore, the sample size is small, and the follow-up time is not long enough. In the subsequent research, we will also focus on the problems caused by peri-implant inflammation and crown-to-root ratio imbalance.

## Materials and methods

### Patients

Forty-one cases with mandibular ameloblastoma underwent partial mandibulectomy and repair utilizing the fibular flap technique. This was performed in the oral and maxillofacial surgery department of the first affiliated hospital of Zhengzhou university from October 2012 to July 2017. There were 26 males and 15 females. The age ranged from 18 to 58 years old, with an average of 34.8 years old. The inclusion criteria were as follows: (1) preoperative design was carried out using 3D printing technology and digital technology, intraoperative resection of the diseased mandible was performed, and the fibular flap technique was used for reconstruction and repair at the same time. (2) No serious complications after the operation. (3) No recurrence of the primary disease. (4) Implant repair was performed at 6–9 months after surgery. The observation period was longer than 24 months.

### Sequential treatment steps

**Figure 2:**
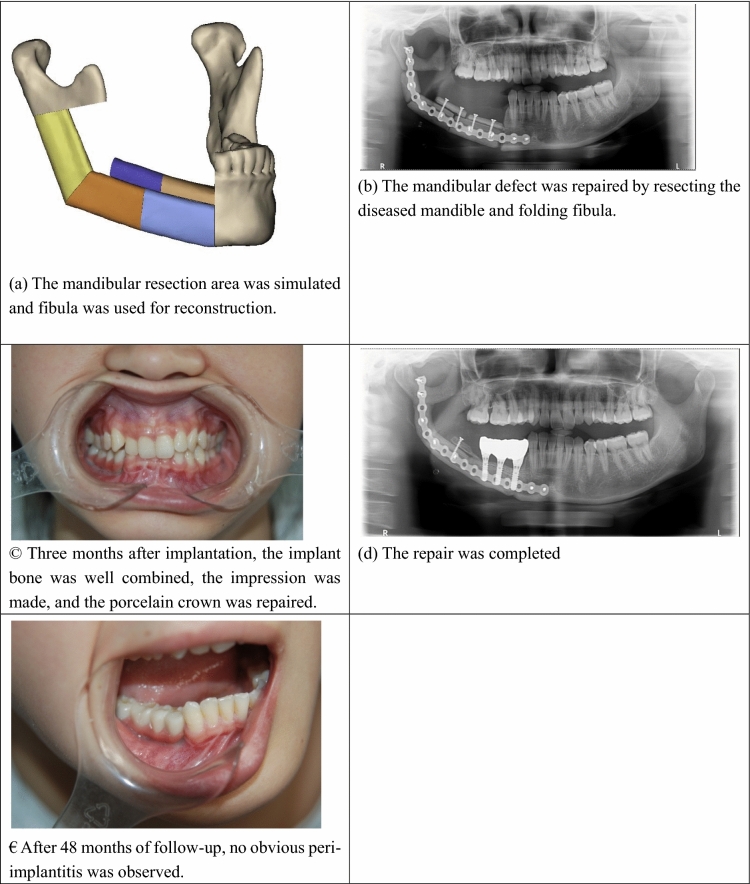
(**a**) The mandibular resection area was simulated and fibula was used for reconstruction. (**b**) The mandibular defect was repaired by resecting the diseased mandible and folding fibula. (**c**) Three months after implantation, the implant bone was well combined, the impression was made, and the porcelain crown was repaired. (**d**) The repair was completed. (**e**) After 48 months of follow-up, no obvious peri-implantitis was observed.

The CT data of the patient’s maxillofacial region and legs which has been obtained before the surgery has been imported into Mimics Research 20.0 software (Belgium Materialise company, https://www.materialise.com/) to complete the 3D reconstruction of the jaw. The scope of resection was clarified when the extent affected by tumor was determined, and then the digital technology was employed to simulate partial resection of the mandible. The mandible date of the healthy side was copied to the affected side by mirroring method and its position was adjusted appropriately. Then after importing the patient’s fibula data, the fibula was cut and shaped according to the best shape of the defect area to be restored. Finally, a 3D head mold was printed, and the titanium plate was pre-bent on the head mold. The operation was carried out in two groups at the same time: one group received a partial mandibulectomy according to the lesion range, retaining the articular disc. The condyle was retained depending on the situation with work on the arteriovenous system carried out in the receiving area. In the other group, the Henry approach was adopted in the posterolateral part of the calf, and the fibular flap was made conventionally. Depending on the defect range, the skin island and fibular length of the free fibular composite flap and the required muscles were designed according to the preoperative digital design scheme. The fibular flap was fixed with a Swiss sinins AO 2.0 prefabricated titanium plate before the pedicle fracture. The branches of the peroneal artery and the external carotid artery were manually sutured with 8-0 Prolene line, and then the peroneal vein was anastomosed with the internal (external) jugular vein with a microvascular anastomosis device to reconstruct blood circulation. The Straumann implant system was used to implant 4.1 standard neck implants at the level of soft tissue through fixed points and stage by stage holes. The length was 10–14 mm and the smooth neck was located at the top of the alveolar crest. Porcelain crown restoration was carried out 3 to 4 months after implantation (Fig. 2).

### Questionnaire and collection

SF-36 and UW-QOL are available in Chinese and have been validated for a Chinese population.

1. SF-36 is a widely used QOL measurement tool to assess the impact of overall health status on QOL. This questionnaire has been used by scholars to evaluate the QOL of cancer patients in China. It mainly includes 9 aspects of physiological function, body pain, physical role, general health, vitality, emotional role, social function, health change and psychological health. The maximum score of each item is 100. The higher the score is, the better the status is.
2. UW-QOL is a self-administered questionnaire that designed for patients with head and neck cancer. Internationally it is one of the most commonly used head and neck cancers QOL questionnaires in use. It includes pain, appearance, activity, entertainment, swallowing, chewing, language, mouth opening, taste, saliva, mood, anxiety, and overall well-being. The domains are scored on a scale ranging from 0 (worst) to 100 (best). Chewing, swallowing, speech, taste and saliva can be used to assess the patient’s oral function.

### Assessment methods

SF-36 and UW-QOL questionnaires were used in the survey, and a combination of doctor’s examinations and questionnaire were adopted in the methodology. Before the operation, the survey (utilizing both types of assessment questionnaires were explained to the patients in full detail. The patients were then instructed to fill in the first questionnaire pre operation, with the second and third surveys to be filled in at 6- and 24-month post operation, respectively. A scoring manual provided by the designer of the questionnaire was used for scoring patient symptoms.

### Statistical analysis

SPSS 20.0 statistical software was used to conduct statistical analysis on the base data of the two groups of patients. Independent sample *t* test was conducted for sf-36 and UW-QOL scores at two time points in each group.

### Ethical approval

This study was conducted under the regulations and guidelines in accordance with institutional research ethics board at The First Affiliated Hospital of Zhengzhou University, Zhengzhou, China (2012-KY-230) and with the 1964 Helsinki declaration and its later amendments or comparable ethical standards. All methods and experimental protocols were reviewed and approved by the research ethics board prior to initiating the study. Informed consent for participation and publication of the images has been obtained from all the participants.
